# Evaluation of baseline pediatric readiness of emergency departments in Manitoba, Canada

**DOI:** 10.1186/s12245-022-00462-0

**Published:** 2022-10-10

**Authors:** Alex Aregbesola, Oana Florescu, Clara Tam, Amanda Coyle, Lisa Knisley, Kaitlin Hogue, Darcy Beer, Scott Sawyer, Terry P. Klassen

**Affiliations:** 1 The Children’s Hospital Research Institute of Manitoba, John Buhler Research Centre, 513-715 McDermot Avenue, Winnipeg, MB R3E, 3P4 Canada; 2 Department of Pediatrics and Child Health, Rady Faculty of Health Sciences, Max Rady College of Medicine, University of Manitoba, Winnipeg, MB Canada; 3 Faculty of Medicine and Dentistry, University of Alberta, Edmonton, Canada

**Keywords:** Pediatric readiness, Survey, Scores, Manitoba, Canada

## Abstract

**Background:**

Data on the readiness of the general emergency departments (EDs) in Canada to care for children requiring emergency care are limited. Recent evidence suggests an inverse association between pediatric readiness of the general ED and mortality.

**Objectives:**

To assess the baseline pediatric readiness of the general EDs in the province of Manitoba, Canada, to care for acutely ill and injured children.

**Methods:**

This was a cross-sectional survey study conducted between 2019 and 2020. We used a validated pediatric readiness research checklist to obtain information on the six domains of the general EDs in Manitoba in the fiscal year 2019. A general ED that managed acutely ill patients (0–17th birthday), except for psychiatric cases (up to the 18th birthday), was defined as eligible. We performed a descriptive analysis using the weighted pediatric readiness score (WPRS) based on a 100-point scale. The factors associated with the total WPRS were examined in linear regression models.

**Results:**

Of the 42 eligible general EDs, 34 centers participated with a participation rate of 81%. However, only 27 general EDs plus one specialized children ED (28, 67%) completed the survey. The overall median WPRS (/100) attained by the general EDs was 52.34 (interquartile range [IQR] = 10.44). The only specialized children ED in Manitoba achieved a score of 89.75. Over half (15, 55.6%) of the general EDs scored 50 or more. The mean volume of the general ED that participated was 4010.9 (± SD 2137.2) pediatric general ED visits/year. The average scores attained in the domains such as coordination of patient care, general ED staffing and training, and quality improvement were low across the five Regional Health Authorities. The general ED volume was directly associated with the total WPRS, regression coefficient, *β* = 0.24 (95% CI 0.04–0.44). Neither the capacity of the general ED to receive pediatric patients from a nursing station, *β* =  − 0.07 (95% CI − 0.28–0.14), nor the capacity to admit pediatric patients that visited the general ED, *β* =  − 0.03 (− 0.23–0.17) was associated with the total WPRS.

**Conclusions:**

The pediatric readiness of the general EDs across Manitoba is comparable to other Canadian region, yet some domains need to be improved.

**Supplementary Information:**

The online version contains supplementary material available at 10.1186/s12245-022-00462-0.

## Introduction

Many general emergency departments (EDs) care for acutely ill and injured children. However, the healthcare providers in the general EDs have expressed concerns about maintaining pediatric expertise and competencies, especially if they do not often treat children or certain pediatric conditions [1]. It is well-established that a delay in prompt response to emergencies may lead to fatal outcomes, heightened in children due to their unique physical and psychosocial needs [2]. The requirements to manage pediatric emergencies differ from adults because of their unique needs in medication, equipment, staff, and pediatric-specific policies and protocols [3]. A low level of pediatric readiness in the general EDs puts children at risk when immediate care is required and may contribute to inconsistencies in the delivery of pediatric emergency care [4]. The National Hospital Ambulatory Medical Care Survey of the United States reported that 20% of the over 141 million annual general ED visits are from children younger than 15 years of age [5]. Likewise, the majority (85%) of children who require emergency care in Canada receive care in the general EDs [1].

To help assess the readiness of the general EDs to care for acutely ill children, the Emergency Medical Services for Children designed a program called the National Pediatric Readiness Project (NPRP) in 2011 as a quality improvement campaign across the general EDs in the United States [5]. They developed a survey that assessed six domains of the general EDs, using weighted pediatric readiness scores (WPRS) to measure the level of readiness of the general EDs to manage acutely ill and injured children. This research checklist is known as the pediatric readiness quality improvement assessment (PRQIA) survey of the general EDs [6].

The PRQIA survey is widely used in the United States to report the pediatric readiness of the general EDs [7–10]. Other countries in Europe [11] and Asia [12] have also adopted the survey. It is gradually gaining momentum in Canada as a valuable tool to assess the readiness of the general EDs [13]. A study from the province of Alberta [13] and our study are the two studies that have completed the baseline pediatric readiness assessments of the general EDs to date in Canada. The Alberta study [13] found an overall WPRS of 48.4/100 in 2019 and concluded that there is an urgent need to improve readiness to respond to high acuity pediatric emergencies in the province. A similar study is ongoing in Ontario, Canada, which shows how provinces in Canada are responding to the need to assess the general EDs to care for acutely ill children.

Relatively recent studies from the USA [14–16] and Europe [17] have shown an inverse association between WPRS of the general EDs and mortality in children. Likewise, the benefits of different interventional measures to increase WPRS to help improve the quality of care in the general EDs have been reported [18–20]. To date, there is no data on the pediatric readiness of the general EDs in Manitoba, Canada. The data is essential to help identify the gaps in providing optimal emergency care for children in the general EDs.

Following a wide spectrum of the usefulness of the PRQIA survey, we used it to examine the baseline pediatric readiness of the general EDs to care for acutely ill and injured children across the province of Manitoba, Canada. This study can help guide the design of interventional measures to address gaps in the delivery of pediatric emergency care. It also has the potential to help advocate for change in policies that can be beneficial to improving pediatric emergency care in the general EDs.

## Methods

### Study design, setting, and population

We conducted a cross-sectional survey sampling of 34 general EDs in the province of Manitoba, Canada, between 2019 and 2020. The general EDs in Manitoba are managed by five Regional Health Authorities (RHA), including the Northern RHA, Southern RHA, Prairie Mountain RHA, Interlake-Eastern RHA, and Winnipeg RHA. We also sampled the only specialized children’s hospital ED in Manitoba to serve as a local reference for this study. An ED was eligible if it provided emergency care for pediatric patients (defined as individuals from 0 up to the 17th birthday in Manitoba), except for psychiatry/mental health cases, which is up to the 18th birthday.

### Online survey

This is a 133-question survey originally developed and validated by the NPRP and the Provincial Council for Maternal and Child Health (PCMCH) and modified to accommodate the uniqueness of Manitoba’s population and geography (supplementary material 1). The survey collected information about six main domains of the ED in the fiscal year 2019. These are coordination of patient care; ED staffing and training; quality improvement; patient safety; policies and procedures; and availability of pediatric equipment/supplies. A detailed description of each domain is available in the survey (supplementary material 1). The survey also collected information about the demographics of the general EDs. The online survey was delivered to the respective general EDs via the Research Electronic Data Capture (RedCap) system [21]. The lead representative at the local general ED coordinated data collection and entry. The primary outcome was the overall WPRS normalized on a 100-point scale. The distribution of WPRS points by the domain of the general EDs is as follows: coordination of patient care is allocated 19 points; ED staffing and training 10 points; quality improvement 7 points; patient safety 14 points; policies and procedures 17 points; and availability of pediatric equipment/supplies 33 points.

We sent out electronic posters (that contained a brief description of the study) via email to all eligible general EDs to obtain expression of interest. Meetings also took place with each RHA to explain the study and seek their support to contact the general EDs and feedback on communication strategies. This was followed by two letters. The first letter was to officially invite the general EDs that expressed interest in participating in the study. The second letter was to inform the general EDs of the details and the instructions needed to complete the survey. We piloted the study in nine general EDs for completeness and usability within the Manitoba context. The first wave of survey distribution was in August 2020. We sent out a scheduled biweekly reminder to complete the survey. This was later changed to weekly towards the end of data collection, which was December 2020. The research team actively followed up with each center by emails and phone calls to inquire and respond to any concerns or challenges in completing the survey. We sent a customized report to each general ED after completing the survey. The report included the WPRS of each general ED, the overall WPRS, and the highest and lowest WPRS recorded in the province of Manitoba. The scores per each of the six domains were also presented in the report. We also included the gaps identified in each general ED that would require attention.

### Sample size and operational approvals

We used convenient sampling based on previous similar successful studies [12, 13, 22]. Both NPRP and PCMCH gave permission to use the survey in Manitoba. The University of Manitoba Health Research Ethics Board approved this study. We also obtained operational approvals from the five RHAs in Manitoba.

### Data analysis

We performed a descriptive analysis to compare the WPRS achieved by the general EDs and the scores in each of the six domains of the general ED by RHA. Data were presented as median scores with an interquartile range and mean scores with standard deviation. We investigated in univariate and multivariable-adjusted linear regression models factors reported in the literature that may influence the overall WPRS. We tested factors such as the general ED volume (pediatric general ED visits/year), the capacity to receive pediatric patients from a nursing station (which is a health care clinic, usually located in the northern isolated communities in Manitoba, where the majority of care is provided by nursing personnel (yes, %)), and the capacity to admit pediatric patients that visited the general ED (yes, %) [23]. The general ED volume was categorized into low volume (1–4999), medium volume (5000–9999), and high volume (≥ 10,000). The total WPRS was log-transformed and treated as a continuous variable. For ethical reasons, we de-identified data for analysis and reporting. We performed data analysis using Stata (v. 16.1; StataCorp, College Station, TX, USA), and the test for statistical significance (at *P* < 0.05) was two sided.

## Results

**Fig. 1:**
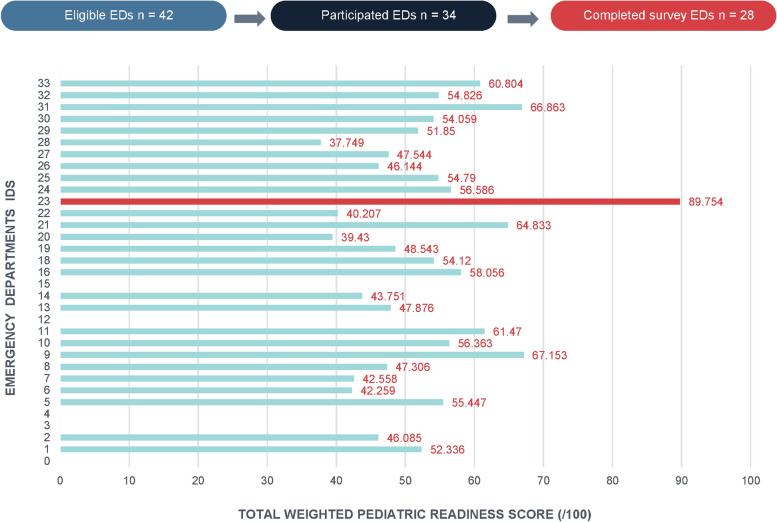
The total WPRS of the general EDs and the specialized children ED in Manitoba. The horizontal lines in green represent the total weighted pediatric readiness scores (WPRS) achieved by the general emergency (EDs) in Manitoba. The horizontal line in red represents the total WPRS achieved by the only specialized children ED in Manitoba. The red line shows the local reference score. The general EDs were de-identified and presented as numbers in no particular order on the *y*-axis. The *x*-axis shows the WPRS normalized on a 100-point scale

We had 34 of the 42 eligible EDs participate in the study, with a participation rate of 81%. However, only 27 general EDs plus one specialized children ED (28, 67%) completed the survey (Fig. 1). The overall median WPRS (/100) attained by the general EDs was 52.34 (interquartile range [IQR] = 10.44). The highest versus lowest total WPRS achieved among the general EDs in Manitoba was 67.15 versus 37.75. The only specialized children ED in Manitoba achieved a score of 89.75. Over half (15, 55.6%) of the general EDs that participated scored of 50 or more. The baseline characteristics of the general EDs by the RHA are presented in Table 1. The mean volume of the general ED by RHA was 11,415.6 (± SD 10,917.5) pediatric general ED visits/year, while the mean volume of all the general ED that participated was 4010.9 (± SD 2137.2). Most of the general ED that participated were classified as low volume (*n* = 21, 91.3%). While 12 (44.4%) general EDs had transfer guidelines to other centers in place for pediatric trauma patients, only 5 (18.5%) could manage pediatric trauma patients in the general ED once they were stabilized. Only 8 (29.6%) of the general EDs received pediatric patients sent directly from a nursing station in a nearby First Nations community. The average scores achieved per each of the domains for the general EDs are presented in Table 2. The average scores attained in the domains such as coordination of patient care, general ED staffing and training, and quality improvement were low across all the RHAs. However, the average scores in patient safety and availability of pediatric equipment/supplies were high. Although there was a disproportional representation of the general EDs by RHA, almost all the RHAs had a total WPRS of over 50.

**Table 1:** Characteristics of emergency departments in Manitoba by Regional Heath Authority between 2018 and 2019

**Characteristics**	**All RHAs** ***n* = 27**	**RHA A** ***n* = 3**	**RHA B** ***n* = 7**	**RHA C** ***n* = 7**	**RHA D** ***n* = 9**	**RHA E** ***n* = 1**
Does your general ED receive pediatric patients that are sent directly from a nursing station in the First Nations community (ies)? Yes (%)	8 (29.6)	3 (100.0)	0 (0.0)	1 (14.3)	4 (44.4)	0 (0.0)
Does your general ED have year-round road access to/from the First Nations community (ies)? Yes (%)	8 (29.6)	2 (66.7)	1 (14.3)	3 (42.9)	1 (11.1)	1 (100.0)
Total number of adult and pediatric ED visits FY 2018/2019 (medical and mental health/substance use visits combined), mean (± SD)	11,415.6 (10,917.5)	NR	11,820.1 (10,077.2)	8866.0 (8821.0)	9135.8 (12,924.9)	33,000 (0.0)
^a^Total number of pediatric ED visits for FY 2018/19 for patients whose medical health issue was the primary reason/diagnosis for the ED visits mean (± SD)	1744.9 (1623.0)	3592.5 (1536.5)	2382.4 (2148.0)	1161.3 (829.5)	762.4 (804.9)	2000 (0.0)
Are pediatric patients who present to the general ED with medical health issues admitted to your hospital? Yes (%)	18 (66.7)	3 (100.0)	7 (100.0)	3 (42.9)	5 (55.6)	0 (0.0)
^a^Number of pediatric patients transferred to another hospital for admission whose medical health issue was the primary reason for the transfer (2018/19), mean (± SD)	26.4 (18.8)	NR	NR	32 (12.7)	9 (5.7)	50(0.0)
Does your general ED manage pediatric trauma patients in the ED once they are stabilized? Yes (%)	5 (18.5)	1 (33.3)	1 (14.3)	1 (14.3)	2 (22.2)	0 (0.0)
Does your general ED have transfer guidelines/protocols in place for pediatric trauma patients? Yes (%)	12 (44.4)	0 (0.0)	3 (42.9)	3 (42.9)	6 (66.7)	0 (0.0)

*ED* Emergency department, *FY* For year, *n* Number of Emergency Department, *NR* Not reported, *RHA* Regional Health Authority, *SD* Standard deviation

^a^Excludes general ED visits or transfers where any of the following Comprehensive Ambulatory Classification System (CACS) codes are used as the primary code: B055 (Mental Health Intervention and Other Counselling), B170 (Mental Health & Psychosocial Condition), or E702 (Other Mental Health Disorder)

**Table 2:** Weighted pediatric readiness scores by domain of the general emergency department for each of the Regional Health Authorities

**Domains**	**Maximum scores achievable per domain of ED**	**RHA A** **(EDs = 3)**	**RHA B** **(EDs = 7)**	**RHA C** **(EDs = 7)**	**RHA D** **(EDs = 9)**	**RHA E** **(EDs = 1)**
Coordination of patient care	19.0	0.0	2.7	2.7	3.2	9.5
ED staffing and training	10.0	0.0	0.7	1.4	0.6	0.0
Quality improvement	7.0	2.0	2.4	0.7	0.0	0.0
Patient safety	14.0	11.4	11.1	12.5	11.5	10.5
Policies and procedures	17.0	3.2	6.3	9.8	9.6	2.1
Availability of pediatric equipment/supplies	33.0	32.8	27.6	27.9	26.0	30.2
Total WPRS	100.0	49.5	50.7	55.0	50.9	52.3

The data presented are average scores in each domain of the general emergency department (ED) by Regional Health Authority (RHA). *WPRS* Weighted pediatric readiness scores

In univariate analysis, the general ED volume was directly associated with the total WPRS, regression coefficient, *β* = 0.27 (95% CI 0.03–0.38) (Table 3). The association was slightly attenuated in multivariable regression models, *β* = 0.24 (95% CI 0.04–0.44). Neither the capacity of the general ED to receive pediatric patients from a nursing station, *β* =  − 0.07 (95% CI − 0.28–0.14), nor the capacity to admit pediatric patients that visited the general ED, *β* =  − 0.03 (− 0.23–0.17) was associated with the total WPRS.

**Table 3:** Association between general ED characteristics and the overall weighted pediatric readiness scores in linear regression models

**Characteristics**	**Univariate linear regression** ***β* (95% CI)**	**Multivariable linear regression** ***β* (95% CI)**
General ED volume	0.27 (0.03–0.38)	0.24 (0.04–0.44)
Capacity to receive pediatric patients from the nursing station	0.020 (− 0.13–0.17)	− 0.07 (− 0.28–0.14)
Capacity to admit pediatric patients that visited the general ED	− 0.03 (− 0.19–0.14)	− 0.03 (− 0.23–0.17)

*ED* Emergency department

## Discussion

This study reports on the online assessment of the pediatric readiness of the general EDs in Manitoba in 2019 and 2020. We observed that the overall WPRS of the general EDs in Manitoba was comparable to the previous Canadian study from the province of Alberta. Over half of the general EDs that participated achieved a score of 50 or more. The WPRS was uneven across the domains of the general ED. On average, the general EDs scored highly in patient safety and availability of pediatric equipment/supplies. However, the scores were low in the coordination of patient care, general ED staffing and training, and quality improvement.

Our study is the second study to publish the baseline WPRS of the general EDs in Canada after the Alberta study. While the Alberta study sampled 59 general EDs and achieved a total WPRS of 46.6, we sampled 27 general EDs and achieved a total WPRS of 52.3 One important reason for assessing the baseline scores of these general EDs is to identify gaps in delivering optimal care and the opportunity to design an intervention to improve the overall WPRS. Studies have shown that compliance with some of the components of the Pediatric Readiness Initiative caused an increase in the median WPRS from 55/100 in 2003 [24, 25] to 68.9/100 in 2013 among the general EDs in the USA [26].

A significant gap was found in pediatric care coordination, general ED staffing and training, and quality improvements of the six domains examined. In keeping with the Alberta study [13] that observed a significant gap in pediatric care coordination and quality improvement, the general EDs in Manitoba also performed poorly in these two domains. The gap in pediatric care coordination was not only recorded in the Canadian general EDs. Boggs et al. [27] reported that only 763 (17.2%) general EDs in the United States had at least one pediatric care coordination among the 4443 (83%) general EDs sampled using the 2015 data.

We examined our data in linear regression models for factors such as the general ED volume and the capacity to admit pediatric patients that visited the general ED, which have been reported in literature to be linked to the overall WPRS [23]. While the volume of the general ED was directly associated with the overall WPRS, we found no association between the capacity to admit pediatric patient that visited the general ED and the overall WPRS.

## Strengths and limitations

The strengths of this study include the use of a validated tool with a wide application across population samples [7–12]. We pilot tested this survey, allowing the opportunity to receive feedback on areas that needed clarification related to the Manitoba context. One of the limitations of this study is that the survey was self-reported and may be affected by an over or under-representation of information or data supplied by the different general EDs. However, we had discussions with the lead representatives for each of the general EDs on where to obtain the information or data needed to complete the survey. The data to complete the survey are available in the different provincial data repositories. Another limitation is the small sample size of the general EDs that participated, which did not allow for robust and sophisticated analysis. We could not include more general ED characteristics in the adjusted regression models or stratify our analysis by the categories of the general ED volume. This study was conducted during the covid-19 pandemic, which may have influenced the participation rate but not the overall WPRS because the data collected used in this study were from the fiscal year 2019. The findings of no association between some of the general ED characteristics and the total WPRS should be treated with caution due to the small sample size.

## Conclusions

In conclusion, the pediatric readiness of the general EDs across Manitoba is comparable to other Canadian region, with an urgent need to improve pediatric care coordination, general ED staffing and training, and quality improvements. These data suggest that the general ED volume is strongly associated with the overall WPRS. In keeping with the quality improvement goals of the NPRP [5], this study has highlighted the domains of the general EDs in Manitoba that require urgent attention to provide optimal care for children requiring emergency care. Areas of future research would include an assessment of the pediatric readiness of centers within the nursing stations and interventional studies looking at improving the total WPRS of the general EDs in Manitoba.

## Supplementary Information

**Additional file 1:** **Supplementary material 1.** Pediatric Readiness Initiative for Emergency Departments Survey.

## Data Availability

Restrictions apply to the availability of these data, which were used under the permission of Shared Health and the five Regional Health Authorities in Manitoba. Data are available from the authors with the permission of Shared Heath and the five Regional Health Authorities in Manitoba.

## Abbreviations

ED: Emergency department
IQR: Interquartile range
NPRP: National Pediatric Readiness Project
PRQIA: Pediatric readiness quality improvement assessment
PCMCH: Provincial Council for Maternal and Child Health
RedCap: Research Electronic Data Capture
RHA: Regional Health Authority
SD: Standard deviation
WPRS: Weighted pediatric readiness score
