# Supplementary material for: Evaluation of baseline pediatric readiness of emergency departments in Manitoba, Canada
Source: Int J Emerg Med. 2022 Oct 10;15:58. doi: 10.1186/s12245-022-00462-0 (PMC9549829; doi:10.1186/s12245-022-00462-0)
Supplement: Supplementary file 1 — Additional file 1: Supplementary material 1. Pediatric Readiness Initiative for Emergency Departments Survey. [file 12245_2022_462_MOESM1_ESM.pdf]

Resize font:

Returning?

Pediatric Readiness Initiative for Emergency Departments

Welcome and thank you for participating in the Pediatric Readiness Initiative for Emergency Departments (EDs) Research Checklist! With your help, we will be able to make great progress towards the goal of ascertaining the current capacity of Manitoba's EDs in terms of their readiness to serve the health needs of children/youth, and opportunities for enhancing this care. The Manitoba version of the checklist is an adaptation of the survey used by the National Pediatric Readiness Project (NPRP) in the United States and the Provincial Council for Maternal and Child Health (PCMCH), Ontario, Canada.

The PCMCH Emergency Department Pediatric Readiness Checklist is based on work that has been underway for several years in the United States that focused on the improvement of emergency care for children in order to ensure that pediatric patients receive appropriate medical care regardless of where they present: 1. The Emergency Medical Services for Children (EMSC) program, and 2. The NPRP, PCMCH, in consultation with various ED providers in community and tertiary hospitals from across Ontario, along with representatives from the EMSC program, has adapted the NPRP pediatric readiness checklist to fit Ontario's context. In addition, an expert panel was convened by PCMCH to develop questions related to children/youth who present to EDs with mental health/substance use issues in Ontario, and feedback was obtained from selected hospitals to ensure the readiness questions fit Ontario's cultural and geographical diversity.

The overall goal is to identify opportunities to improve the emergency system for pediatric patients to ensure they receive optimal care, regardless of their condition or at which ED they present. The questions included in the readiness checklist are related to 6 pediatric-specific domains: 1. Coordination of patient care; 2. ED staffing and training; 3. Quality improvement; 4. Patient safety; 5. Policies and procedures; and 6. Availability of pediatric equipment/supplies. We will leverage the knowledge of TRanslating Emergency Knowledge for Kids (TREKK) in approach/expertise, ED network, and established relationships within the overall healthcare system.

Helpful Information About the Online Tool

- All of the questions in the checklist are mandatory, with the exception of the last two questions. When you are ready to submit your responses at the end, if there are any mandatory questions that have not yet been answered, a window will appear indicating the outstanding questions and their specific number.
- If you have answered a question and then decide you would like to delete your response(s) for that question (for example, one in which several options are possible and have been selected), you can either do this manually or you can click on the "reset" button for that question. Doing so will delete the responses for that question only.
- If you would like to save your answers and complete the readiness checklist at a later time, or if you would like a colleague to review the responses and/or answer some of the questions that have not yet been answered:
- Click on the "Save & Return Later" button (on the last page of the checklist questions) at which time you will receive a "Return Code". **Please remember to save the code before closing the survey page.**
- To re-enter the survey, use the link first received. Click on the box that says "Returning?" which is located in the top right corner of the page. You will then be directed to enter your return code into a box. This will take you back to the survey you have already started to complete.
- To submit your final responses, please click on the "Submit" button (on the last page of the checklist questions). Once that is done, you will not be able to review or change any of your answers.
- Individual hospital responses will be kept confidential. Approximately 6-8 weeks after completion of the readiness checklist, you will receive a score for your hospital's ED regarding its pediatric readiness, along with an analysis of areas for potential improvement - based on your responses. Aggregated and/or anonymized responses from all participating hospitals will help inform a broader implementation strategy and may become part of a report regarding the status of ED readiness in relation to pediatric patients.
- **We would appreciate if you would complete the survey on or before October 31, 2020 16:00 CST**

Please note: To ensure a comprehensive set of responses that are representative of the organization, **only one submission per hospital will be accepted**. It will be up to each hospital to determine who from within the organization should be involved with this process. If you are going to be consulting with a colleague(s) about the responses, or if a colleague(s) is going to be answering some of the questions, it will likely be helpful to print the PDF version of the checklist that was included in the accompanying email. The PDF version includes all of the branching questions which, in the online version, only show up based on the answers provided.

If you have any questions about the Pediatric Readiness Initiative for Emergency Department Checklist or the initiative overall, please contact:

Alex Aregbesola,
aaregbesola@chrim.ca
(204)977-5697

Name:

Title/Position:

Phone Number:

Email:

Hospital Name:

Regional Health Authority (RHA):

- ◯ Winnipeg RHA
- ◯ Southern Health - Sante Sud
- ◯ Prairie Mountain Health
- ◯ Northern Health Region
- ◯ Interlake-Eastern RHA

reset

Notes:

- **Pediatric patients are defined as 0 up to the 17th birthday in Manitoba (except for psychiatry/mental health cases which is up to the 18th birthday); for the purposes of this survey, this same age range will apply to all questions.**
- **The role parents/caregivers/families play in the care of their children is critical when providing care to pediatric patients (depending on age/developmental stage and, if needed, permission to involve others). Family-centered care is crucial to this population and allows for the inclusion of parents/caregivers/families in decision-making relative to patient-care plans; this has been shown to improve patient safety and increase patient and family satisfaction with their medical care/ experience.**

The first few questions have to do with the infrastructure of your hospital, its ED and the pediatric volumes. Please note that questions pertaining specifically to pediatric patients who present with mental health/substance use issues are included in a separate section in a latter part of the readiness checklist.

1. Which of the following is the best description of your ED's configuration for the care of pediatric patients who present with **medical health issues:**

- ◯ Pediatric ED in a **stand-alone tertiary children's hospital**
- ◯ Separate pediatric ED in a combined **Pediatric Academic Health Sciences Centre/Academic Health Sciences Centre**
- ◯ ED in a combined **Pediatric Academic Health Sciences Centre/Academic Health Sciences Centre with pediatric and adult patients seen in the same area**
- ◯ General ED **in a community hospital with separate room/area designated for pediatric patients**
- ◯ General ED in a community hospital with **pediatric and adult patients seen in same area**
- ◯ Other

reset

2. The age range for pediatric patients is 0 up to the 18th birthday. Is this same age range used by your hospital to define pediatric patients who present to the ED?

- ◯ Yes - this age range is used for **pediatric patients who present to the ED with *medical and/or mental health/substance use issues***
- ◯ No - this age range is used **only for pediatric patients who present to the ED with *medical health issues*** (Go to #100)
- ◯ No this age range is used **only for pediatric patients who present to the ED with *mental health issues*** (Go to #3)
- ◯ This age range is **not used** for pediatric patients who present to the ED with ***medical health/substance use issues*** (Go to #3 and #100)

reset

4. Does your ED receive pediatric patients that are sent ***directly from a nursing station*** in a First Nations community?

- ◯ Yes
- ◯ No (Go to #7)

reset

8. Please indicate the ***total number of pediatric ED visits*** for FY 2018/19 for patients whose ***medical health issue*** was the **primary reason/diagnosis** for the ED visits. Note: This would ***exclude*** ED visits where any of the following Comprehensive Ambulatory Classification System (CACS) codes are used as the primary code: B055 (Mental Health Intervention and Other Counselling), B170 (Mental Health & Psychosocial Condition), or E702 (Other Mental Health Disorder).

[ ]

Numeric data only (e.g., 5000) no commas, not "five thousand"

9. Are pediatric patients who present to the ED with ***medical health issues*** admitted to your hospital?

- ◯ Yes
- ◯ No (Go to #12)

reset

This section includes details about how pediatric trauma patients are managed at your hospital.

13. Does your hosptial manage pediatric trauma pateints in the ED ***once they are stabilized***?

- ◯ Yes
- ◯ No (Go to #17)

reset

The following questions pertain to the staffing resources available for the care of children/youth in your hospital's ED, regardless of the presenting issue.

18. Does your hospital have a ***physician coordinator*** who is assigned the role of overseeing various administrative aspects of pediatric emergency care (e.g., overseeing quality improvement, collaborating with nursing staff regarding pediatric initiatives, ensuring opportunities are in place for the development/maintenance of pediatric skills, developing and periodically reviewing policies? ***Note: The pediatric component of the physician coordinator role may be incorporated within the overall physician coordinator role.***

- ◯ Yes - the physician coordinator is responsible for the administrative aspects of ***pediatric emergency care only*** (Go to #20)
- ◯ Yes - the physician coordinator is responsible for the administrative aspects of ***pediatric and adult emergency care*** (Go to #19)
- ◯ No (Go to #21)

reset

25. Which of the following ***life support course*** are required by your hospital as part of ***physician credentialing*** for ED physicians who provide care to children/youth (select all that apply):

- ☐ Basic Life Support Advanced Cardiac Live Support (ACLS)
- ☐ Pediatric Basic Life Support (PBLS) (e.g., Healthcare Provider CPR certification or basic life support)
- ☐ Pediatric Advanced Life Support (PALS)
- ☐ APLS - The Pediatric Emergency Medicine Resource Neonatal Resuscitation Program (NRP)
- ☐ International Trauma Life Support (ITLS; formerly Basic Trauma Life Support)
- ☐ Advanced Trauma Life Support (ATLS)
- ☐ Pediatric Advanced Trauma Support (P-ATLS)
- ☐ Other
- ☐ None of the above

26. Does your hospital require specific **pediatric competency evaluations of physicians** who staff the ED (e.g., sedating and analgesia)?

- ◯ Yes
- ◯ No

reset

27. Does your hospital offer simulation-based education to physicians who staff the ED pertaining to the care of pediatric patients who present o the ED?

- ◯ Yes (Go to #28)
- ◯ No (Go to #30)

reset

30. Thinking of the RNs who staff your ED and care for your children/youth, which of the following are required by your hospital (select all that apply):

- ☐ Continuing education requirements in pediatric emergency care
- ☐ Maintenance of specialty certification for nurses (e.g., CEN, CPEN)
- ☐ Maintenance of Emergency Nursing Pediatric Certification (ENPC)
- ☐ Other
- ☐ None of the above

31. Which of the following ***life support courses*** are required by your hospital ***condition of employment*** OR ***within 6 months of employment*** for ED RNs who provide care to childre/youth (select all that apply):

- ☐ Basic Life Support
- ☐ Advanced Cardiac Life Support (ACLS)
- ☐ Pediatric Basic Life Support (PBLS (e.g., Healthcare Provider CPR certification or basic life support)
- ☐ Emergency Nurse Pediatric Course (ENPC)
- ☐ Pediatric Advanced Life Support (PALS)
- ☐ APLS-The Pediatric Emergency Medicine Resource
- ☐ Neonatal Resuscitation Program (NRP)
- ☐ International Trauma Life Support (ITLS; formerly Basic Trauma Life Support)
- ☐ Trauma Nursing Core Course (TNCC)
- ☐ Other
- ☐ None of the above

32. Does your hospital require specific ***pediatric competency evaluations of RNs*** who staff the ED (e.g., triage, pain assessment)?

- ○ Yes
- ○ No

reset

33. Does your hospital offer ***simulation-based education to RNs*** who staff the ED pertaining to the care of pediatric patients who present o the ED?

- ○ Yes (go to #34)
- ○ No (go to #36)

reset

51. Do staff and physicians who staff the ED access ***external evidence-based/evidence-informed resources*** related to the diagnosis and management of pediatric emergency conditions?

- ○ Yes
- ○ No

reset

52. If yes, please indicate the source for these resources (select all that apply):

- ☐ TREKK (TRanslating Emergency Knowledge for Kids
- ☐ Other resource(s)

53. Does your hospital provide training/have access to training for ED staff regarding the provision of ***child/family-centred care***?

- ○ Yes
- ○ No (Go to #55)

reset

Pediatric Patient Safety/Quality Improvement in the ED

59. Does your ED have a ***pediatric patient care-review process***?

Note: This may be a separate Quality Improvement/Performance Improvement Plan for pediatric patients or it may be integrated into the overall ED Quality Improvement/Performance Improvement Plan.

- ○ Yes
- ○ No (Go to #61)

reset

61. Are all pediatric patients who are seen in the ED weighted in kilograms (without conversion from pounds)? ***Note: This includes critical situations when a child might bypass triage and have his/her weight estimated in kilograms.***

- ○ Yes (Go to #62)
- ○ No (Go to #63)

reset

65. Is blood pressure monitoring available for children of all ages/sizes who are seen in the ED, based on severity of illness?

- ◯ Yes
- ◯ No

reset

66. Is pulse oximetry monitoring available for children of all ages who are seen in the ED, based on severity of illness?

- ◯ Yes
- ◯ No

reset

67. Is a written procedure in place for notification of physicians when abnormal vital signs are found in all pediatric patients who are seen in the ED?

- ◯ Yes
- ◯ No

reset

68. Is a process in place for the use of pre-calculated drug dosing in all pediatric patients who are seen in the ED (e.g., IV drug library, order sets, etc.)?

- ◯ Yes
- ◯ No

reset

69. Does your ED have a room for children/youth who would benefit from a quiet environment/reduced stimulation (i.e., patients with developmental disabilities)?

- ◯ Yes
- ◯ No

reset

70. Is there a process in place that allows for 24/7 access to interpreter services in the ED?

- ◯ In-person interpretation services
- ◯ Telephone language line in-person sign language interpretation serves
- ◯ TTY device or other assistive technology products for the deaf, hard of hearing, and hearing impaired
- ◯ None of the above

reset

The following section deals with policies and/or procedures that your ED has in place to address the needs of children/youth; these pediatric policies may be integrated into the overall ED policy manual or may be listed separately. They should also be written and available to staff and physicians in the ED.
Triage/Assessment

71. Does your ED have a triage policy that specifically addresses ill and injured children/youth, ***including*** children youth who present with mental health/substance use issues?

- ◯ Yes (Go to #72)
- ◯ Triage policy in place; does not include children/youth who present with mental health/substance use issues (Go to #72)
- ◯ No (Go to #73)

reset

74. When children/youth present to your ED, does the triage process include a basic mental health assessment or is the mental health assessment deferred until the child/youth is seen in a room by a member of the ED team (select all that apply):

- ☐ Part of the triage process
- ☐ Deferred until the patient is in a room
- ☐ Unsure

75. If a pediatric patient in the ED is perceived by staff as ***having needs that would benefit from incorporating a cultural perspective into the assessment and/or management while in the ED***, does your hospital have access to, and utilize, such resources (e.g., culture-specific patient care navigators, elders from the community, traditional healers, cultural/spiritual ceremonies, etc.)?

- ◯ Yes
- ◯ No (Go to #77)

reset

77. Does your hospital have ***culture-specific pathways*** in place regarding the care provided to paediatric patients ***in the ED***?

- ◯ Yes
- ◯ No (Go to #81)

reset

Policies and Procedures

81. Thinking of pediatric patients who present with ***medical health issues***, please indicate if your ED has policies and procedures for the following (select all that apply):

- ☐ Pediatric patient assessment and reassessment
- ☐ Immunization assessment and management of the under-immunized child
- ☐ Child maltreatment
- ☐ Death of the child in the ED
- ☐ Educed-dose radiation for CT and x-ray imaging based on pediatric age or weight
- ☐ None of the above

82. Does your ED have a policy for promoting family-centred care? (e.g., family presence, family involvement in clinical decision-making, etc.)

- ◯ Yes
- ◯ No

reset

83. Does your hospital's disaster plan address issues specific to the care of children/youth?

- ◯ Yes
- ◯ No

reset

The following section pertains to your hospital's inter-facility transfer guidelines.

84. Thinking about pediatric patients who present to your hospital's ED, does your hospital have written ***interfacility guidelines or agreements*** that outline procedural and administrative policies with other hospitals for the transfer of patients of all ages, including children/youth in need of care not available at your hospital?

- ◯ Yes - Inter-facility transfer guidelines/agreements exist and ***are the same*** for patients with medical and mental health/substance use issues (Go to #85)
- ◯ Yes - Inter-facility transfer guidelines/agreements exist and ***are different*** for patients with medical versus mental health/substance use issues (Go to #86 and #127)
- ◯ Yes- - Inter-facility transfer guidelines/agreements exist ***only*** for patients with ***medical health issues*** (Go to #87 and #128)
- ◯ Yes - Inter-facility transfer guidelines/agreements exist ***only*** for patients with ***mental health/substance use issues*** (Go to #88 and #127)
- ◯ No - Inter-facility transfer guidelines/agreements ***do not exist*** for patients with medical health or mental health/substance use issues (Go to #88 and #128)

reset

The next section has to do with pediatric-specific equipment, supplies, and medication in your ED and how these are stored and re-supplied.
Note: if you have not already printed the entire assessment, we recommend that you print this portion and use it when determining which pieces of pediatric-specific equipment/supplies are available in your ED.

89. Are safe pediatric crib/beds available in the ED?

- Yes
- No

reset

90. Are all the ED staff and physicians trained on the location of all pediatric equipment and medications?

- Yes
- No

reset

91. Is there a daily method used to verify the proper location and function of pediatric equipment and supplies?

- Yes
- No

reset

92. Is a medication chart, length-based (Broselow) tape, medical software, or other system readily available to ensure proper sizing of resuscitation equipment and proper dosing of medication?

- Yes
- No

reset

93. Is the pediatric resuscitation equipment stored on a separate pediatric cart (i.e., Pediatric Broselow Chart)?

- Yes
- No

reset

94. Are each of the following ***monitoring equipment items*** available for immediate use in the ED?

	Yes	No	
Neonatal blood pressure cuff	○	○	reset
Infant blood pressure cuff	○	○	reset
Child blood pressure cuff	○	○	reset
Defibrillator with paediatric and adult capabilities including pads/paddles	○	○	reset
Pulse oximeter with paediatric and adult probes	○	○	reset
Continuous end-tidal CO2 monitoring device	○	○	reset

95. Are each of the following ***fluid resuscitation equipment items*** available for immediate use in the ED?

	Yes	No	
22 gauge catheter-over-the-needle	○	○	reset
24 gauge catheter-over-the-needle	○	○	reset
Paediatric intra-osseous needles (EZ-10)	○	○	reset
Paediatric intra-osseous needle gun	○	○	reset
IV administration sets with calibrated chambers and extension tubing and/or infusion devices with ability to regulate rate and volume of infusate	○	○	reset
Umbilical vein catheters (3.5F or 5.0F)	○	○	reset
Central venous catheters (any two sizes in range, 47F)	○	○	reset

Blood	◯	◯	reset
Blood Products	◯	◯	reset

96. Are each of the following ***respiratory/airway/trauma management equipment items*** available for immediate use in the ED?

	Yes	No	
Endotracheal tubes: cuffed or uncuffed 2.5 mm	◯	◯	reset
Endotracheal tubes: cuffed or uncuffed 3.0 mm	◯	◯	reset
Endotracheal tubes: cuffed or uncuffed 3.5 mm	◯	◯	reset
Endotracheal tubes: cuffed or uncuffed 4.0 mm	◯	◯	reset
Endotracheal tubes: cuffed or uncuffed 4.5 mm	◯	◯	reset
Endotracheal tubes: cuffed or uncuffed 5.0 mm	◯	◯	reset
Endotracheal tubes: cuffed or uncuffed 5.5 mm	◯	◯	reset
Endotracheal tubes: cuffed 6.0 mm	◯	◯	reset
Laryngoscope blades: straight, size 00	◯	◯	reset
Laryngoscope blades: straight, size 0	◯	◯	reset
Laryngoscope blades: straight, size 1	◯	◯	reset
Laryngoscope blades: straight, size 2	◯	◯	reset
Laryngoscope blades: curved, size 2	◯	◯	reset
Pediatric-sized Magill forceps	◯	◯	reset
Nasopharyngeal airways: infant-sized	◯	◯	reset
Nasopharyngeal airways: child-sized	◯	◯	reset
Oropharyngeal airways: size 0 (50 mm)	◯	◯	reset
Oropharyngeal airways: size 1 (60 mm)	◯	◯	reset
Oropharyngeal airways: size 2 (70 mm)	◯	◯	reset
Oropharyngeal airways: size 3 (80 mm)	◯	◯	reset
Stylets for pediatric/infant-sized endotracheal tubes	◯	◯	reset
Tracheostomy tubes: size 3.0 mm	◯	◯	reset
Tracheostomy tubes: size 3.5 mm	◯	◯	reset

Tracheostomy tubes: size 4.0 mm	◯	◯	reset
Bag-mask device, self-inflating: infant, 450ml	◯	◯	reset
Masks to fit bag-mask device adaptor: neonatal	◯	◯	reset
Masks to fit bag-mask device adaptor: infant	◯	◯	reset
Masks to fit bag-mask device adaptor: child	◯	◯	reset
Clear oxygen masks: standard infant	◯	◯	reset
Clear oxygen masks: standard child	◯	◯	reset
Non-rebreather masks: infant-sized	◯	◯	reset
Non-rebreather masks: child-sized	◯	◯	reset
Nasal cannulas: infant	◯	◯	reset
Nasal cannulas: child	◯	◯	reset
Laryngeal mask airways: size 1	◯	◯	reset
Laryngeal mask airways: size 1.5	◯	◯	reset
Laryngeal mask airways: size 2	◯	◯	reset
Laryngeal mask airways: size 2.5	◯	◯	reset
Laryngeal mask airways: size 3	◯	◯	reset
Suction catheters: at least one in range 6-8F	◯	◯	reset
Suction catheters: at least one in range 10-12F	◯	◯	reset
Supplies/kit for pediatric patients with difficult airways (supraglottic airways of all sizes, needle cricothyrotomy supplies, surgical cricothrotomy kit)	◯	◯	reset
Pediatric cervical collars sized to fit neonates, children, and adolescents (i.e., Aspen collars in sized PD 1-5)	◯	◯	reset
Percutaneous over-the-wire chest tube innsertion kit with sizes 14, 20, and 289 (i.e., Cook Medical Kit)	◯	◯	reset

The following questions have to do with the barriers/challenges that may exist in providing care to children/youth who present to your ED with medical health issues.

97. Please indicate which of the following factors, if any, you perceive as a ***barrier(s) in providing care*** to children/youth who present to your ED with ***medical health issues*** (select all that apply):

- ☐ Cost of personnel
- ☐ Costs associated with training of personnel
- ☐ Lack of physicians trained in pediatrics
- ☐ Lack of nurses trained in pediatrics
- ☐ Lack of administrative support
- ☐ Lack of social work support
- ☐ Lack of policies for pediatric emergency care
- ☐ Lack of a Quality Improvement/Performance Improvement Plan specific to pediatrics
- ☐ Lack of a disaster plan specific to pediatrics
- ☐ Other activities/programs/initiatives etc. within the hospital have a higher priority
- ☐ Lack of educational resources
- ☐ Lack of pediatric guidelines or standardized pediatric pathways
- ☐ Unaware that pediatric guidelines exist and/or unfamiliar with these guidelines
- ☐ Other

This section includes information details about your hospital's infrastructure, patient volumes, staffing, resources, patient safety, triage/assessment, policies, and procedures, inter-facility transfer guidelines, barriers and challenges pertaining to how pediatric patients who present to the ED with mental health/substance use issues.

98. Does your hospital see children/youth in the ED who presents with ***mental health/substance use issues***?

- ◯ Yes
- ◯ No (Go to #132)

reset

132. If you have any additional comments you would like to share about your hospital's readiness to provide care to pediatric patients who present to your ED, please note them here:

Expand

133. If you have any comments about this survey (e.g., ease of understanding the questions, questions you think should be included, etc.) or the mechanism for collecting your responses (i.e., ease in using the online tool), please note them here:

Expand

Thank you very much for completing the Pediatric Readiness Initiative for Emergency Departments Checklist!

Submit

Save & Return Later
